# Effects of Anodal tDCS on Arithmetic Performance and Electrophysiological Activity

**DOI:** 10.3389/fnhum.2020.00017

**Published:** 2020-02-11

**Authors:** Jochen A. Mosbacher, Clemens Brunner, Michael A. Nitsche, Roland H. Grabner

**Affiliations:** ^1^Educational Neuroscience, Institute of Psychology, University of Graz, Graz, Austria; ^2^Department of Psychology and Neurosciences, Leibniz Research Centre for Working Environment and Human Factors, Dortmund, Germany; ^3^Department of Neurology, University Medical Hospital Bergmannsheil, Ruhr-University Bochum, Bochum, Germany; ^4^BioTechMed-Graz, Graz, Austria

**Keywords:** arithmetic, fact retrieval, procedural calculation, EEG, transcranial direct current stimulation

## Abstract

Arithmetic abilities are among the most important school-taught skills and form the basis for higher mathematical competencies. At the same time, their acquisition and application can be challenging. Hence, there is broad interest in methods to improve arithmetic abilities. One promising method is transcranial direct current stimulation (tDCS). In the present study, we compared two anodal tDCS protocols in their efficacy to improve arithmetic performance and working memory. In addition, we investigated stimulation-related electrophysiological changes. Three groups of participants solved arithmetic problems (additions and subtractions) and an n-back task before, during, and after receiving either frontal or parietal anodal tDCS (25 min; 1 mA) or sham stimulation. EEG was simultaneously recorded to assess stimulation effects on event-related (de-) synchronisation (ERS/ERD) in theta and alpha bands. Persons receiving frontal stimulation showed an acceleration of calculation speed in large subtractions from before to during and after stimulation. However, a comparable, but delayed (apparent only after stimulation) increase was also found in the sham stimulation group, while it was absent in the group receiving parietal stimulation. In additions and small subtractions as well as the working memory task, analyses showed no effects of stimulation. Results of ERS/ERD during large subtractions indicate changes in ERS/ERD patterns over time. In the left hemisphere there was a change from theta band ERD to ERS in all three groups, whereas a similar change in the right hemisphere was restricted to the sham group. Taken together, tDCS did not lead to a general improvement of arithmetic performance. However, results indicate that frontal stimulation accelerated training gains, while parietal stimulation halted them. The absence of general performance improvements, but acceleration of training effects might be a further indicator of the advantages of using tDCS as training or learning support over tDCS as a sole performance enhancer.

## Introduction

Basic arithmetic skills belong to the most important skills for educational achievement and for everyday life in general (Neisser et al., 1996; Parsons and Bynner, 1997, 2005). Not only do they allow performing simple calculations, but they also form the basis for higher mathematical competencies and understanding (e.g., Geary, 2011; Price et al., 2013). However, learning and application of arithmetic and mathematical abilities can be challenging. This is especially true for people suffering from developmental dyscalculia, with prevalence rates of 3–6% being one of the most common learning disorders (Shalev et al., 2000). But even in the general population, 22.7% perform at proficiency level 1 or below (being only able to carry out simple mathematical processes) according to an OECD survey of adult skills (OECD., 2016). As deficits in arithmetic and mathematical skills place a large burden on the individual, the interest in means to support and improve these abilities is constantly growing (e.g., Parsons and Bynner, 2005). In this study, we investigated the effects of anodal transcranial direct current stimulation (a-tDCS), a non-invasive brain stimulation method, on arithmetic abilities and related oscillatory brain activity. Performance was assessed prior, during, and after stimulation to investigate stimulation-induced improvements, EEG was recorded concomitantly in order to investigate oscillatory correlates of arithmetic performance and stimulation-induced changes.

On the behavioral level, processing of arithmetic problems relies largely on the application of two sets of knowledge: declarative knowledge about arithmetic facts and procedural knowledge about arithmetic operations (Campbell and Xue, 2001; Grabner and De Smedt, 2011). Small, easy problems (e.g., additions with sums ≤10) are mostly solved by fact retrieval (direct recall of the solution, an arithmetic fact, from memory), reflecting a fast and largely effortless process. Large, more complex problems (e.g., two-digit/two-digit subtractions), in contrast, are primarily solved by the application of procedural strategies (based on knowledge of arithmetic operations), which is slower and more effortful (Campbell and Xue, 2001; Destefano and LeFevre, 2004). For instance, solving a two-digit/two-digit subtraction might involve the breakdown of the problem into smaller steps (56–27 → 56–20 = 36 → 36–6 = 30 → 30–1 = 29). These procedural calculation processes incorporate additional, domain-general functions like working memory (WM) more strongly than fact retrieval processes (Destefano and LeFevre, 2004).

On a neurophysiological level, mental arithmetic requires the interplay of a wide network of brain sites (see Menon, 2015), whereby fact retrieval is accompanied by higher activation in the angular gyrus (AG), while procedural calculation is associated with stronger activation of a broad network, including the dorsolateral prefrontal cortex (DLPFC) and the intraparietal sulcus (IPS; Grabner et al., 2009). Previous EEG research demonstrated a clear distinction between fact retrieval and procedural calculation in oscillatory event-related (de-)synchronization (ERS/ERD) patterns. Already in an early study, higher theta band power in the left hemisphere has been associated with fact retrieval during arithmetic problem solving (Earle et al., 1996). This has later been corroborated by studies showing that the processing of small (fact retrieval) problems was accompanied by stronger left hemispheric theta band ERS, while the processing of larger (procedural) problems led to less theta band ERS, but stronger alpha band ERD, especially over bilateral parieto-occipital areas (De Smedt et al., 2009; Grabner and De Smedt, 2011). Further support for this distinction comes from a training study, showing an increase in theta band ERS and a decrease in lower alpha band ERD with increasing use of fact retrieval over procedural calculation in complex arithmetic problems (Grabner and De Smedt, 2012). Against this background, these regions discussed above have been the targets of most transcranial electrical stimulation (tES) studies on arithmetic performance (for a review, see Schroeder et al., 2017), and theta and alpha band ERS/ERD patterns have been used to investigate physiological stimulation effects (Rütsche et al., 2015).

TES comprises different approaches of non-invasive brain stimulation by weak electric currents (generally 1–2 mA), with tDCS being the most commonly used version. In tDCS, a constant direct current is applied via two or more electrodes (anodes and cathodes). It is assumed that the applied current has mainly excitatory effects on the cortical regions beneath the anode, but primarily inhibitory effects on the regions beneath the cathode on the macroscopic level, if stimulation is conducted within a specific conventional range of stimulation intensity and duration (Nitsche et al., 2008; Paulus, 2011). The first studies on the effects of tDCS on arithmetic performance stimulated parietal sites, either unilaterally or bilaterally (Clemens et al., 2013; Hauser et al., 2013; Kasahara et al., 2013; Klein et al., 2013). Clemens et al. (2013) investigated effects of anodal tDCS (a-tDCS) over the right parietal cortex but could not find any effects on multiplication verification tasks. However, an additional fMRI analysis indicated a stronger activation in the AG after stimulation when processing problems which were rehearsed during tDCS. Using a different approach, Klein et al. (2013) found a reduced distractor distance effect in an addition verification task during bilateral parietal a-tDCS, while cathodal stimulation showed no effects. Hauser et al. (2013) applied a-tDCS between two task sessions and found that stimulation over left parietal regions reduced calculation times in large subtractions, while stimulation over right parietal regions as well as bilateral stimulation showed no effects. Interestingly, these effects do not seem to be limited to subtractions, as left anodal / right cathodal parietal tDCS also improved calculation times in complex multiplications (Kasahara et al., 2013). Hence, the left parietal region seems to be a worthwhile target for a-tDCS in the context of arithmetic processing.

The second promising target for tDCS is the left DLPFC. Anodal stimulation of this region has been found to improve arithmetic verification in a group with high math anxiety (Sarkar et al., 2014) and performance in a serial subtraction task (Pope et al., 2015). However, regarding frontal stimulation it is unclear whether the effects of stimulation are domain-specific or if tDCS affects more domain-general functions like working memory (WM) and only indirectly improves arithmetic performance. TDCS, especially over frontal regions, has been found to boost working memory performance (Zaehle et al., 2011; Brunoni and Vanderhasselt, 2014), and Pope et al. (2015) ascribed the positive effects of left frontal a-tDCS on a serial subtraction task to stimulation-induced improvements of working memory. This would also be in line with one study finding beneficial effects of left frontal a-tDCS on a serial addition task only when the stimulation conducted before the task was accompanied by a difficult WM task (Gill et al., 2015). Disentangling the effects of a-tDCS on working memory and arithmetic abilities is complicated, because working memory is an integral part of arithmetic processes, especially procedural calculation (Destefano and LeFevre, 2004; Kasahara et al., 2013). Additionally, like most treatments tDCS does not only have beneficial effects. In a more recent study expanding on the findings of Hauser and colleagues, Rütsche et al. (2015) found that parietal stimulation might indeed enhance performance in large, complex arithmetic problems, but at the same time, this stimulation protocol impaired performance in small, easy problems. This dissociation of effects was accompanied by differential changes in ERS/ERD patterns. While parietal a-tDCS increased lower-alpha ERD during large, complex problems, it decreased theta band ERS during small problems. Hence, there might be a trade-off between beneficial and detrimental stimulation effects, which could prove problematic for the use of a-tDCS as a means to improve arithmetic performance.

This study was performed to expand on prior work by comparing effects of frontal and parietal a-tDCS on arithmetic performance and assessing changes in WM and concomitant EEG. To this end, participants were asked to solve arithmetic problems similar to those used in prior studies (Hauser et al., 2013; Rütsche et al., 2015; small and large additions and subtractions) before, during, and after receiving a-tDCS to either left frontal (targeting the dorsolateral prefrontal cortex; DLPFC) or left parietal regions (targeting the posterior parietal cortex; PPC), or sham stimulation. Additionally, high-density EEG was recorded concomitantly to investigate stimulation-induced changes in ERS/ERD patterns. A short WM task was administered in each phase to investigate stimulation-induced changes in WM performance and to examine task-specificity of stimulation. Based on prior results, we expected that both active stimulations (left frontal and left parietal a-tDCS) improve procedural calculation, with especially frontal stimulation also boosting WM performance (Zaehle et al., 2011; Hauser et al., 2013; Rütsche et al., 2015). This should be accompanied by changes in ERS/ERD patterns in theta and alpha bands, whereby we expected improvements in procedural calculation to be linked to a reduced ERD in alpha bands. Finally, we were eager to investigate, whether the adverse effects of a-tDCS on fact retrieval processes (Rütsche et al., 2015) would replicate in this study.

## Methods

### Sample

In total, 72 persons, recruited at the University of Graz and via e-mail and social media, participated in this study. All participants were right handed, without prior or current neurologic or psychiatric disorders, drug use, and any medication potentially influencing the state of their central nervous system. Five participants had to be excluded from analyses because of insufficient performance in at least one block of arithmetic problems (no or only one correct trial). Another two participants had to be excluded because they did not conduct the WM task correctly (no correct trials in at least one block). Finally, three participants had to be excluded from the final analysis because the EEG recorded during the stimulation phase could not be analyzed due to of bad data quality. Hence, the final sample consisted of 62 participants, with 21 receiving left frontal a-tDCS, 20 left parietal a-tDCS and 21 sham stimulation (demographic data listed in Table 1). Groups did not differ in arithmetic ability or male/female ratio. However, the group receiving frontal stimulation was significantly younger than the groups receiving parietal or sham stimulation [*F*_(2, 59)_ = 7.376; *p* = 0.001]. All participants were thoroughly informed about the study protocol, stimulations and procedures, and gave written informed consent. For participation, they received either 20 € or a study-participation certificate for course credits of 3.5 h. The study was approved by the ethics commission of the University of Graz (No: 60–2015/16).

**Table 1:** Demographic data and basic arithmetic ability.

	**Total (*****N*** **=** **62)**		**Stimulation groups**					
			**Frontal (*****N*** **=** **21)**		**Parietal (*****N*** **=** **20)**		**Sham (*****N*** **=** **21)**	
	***M***	***SD***	***M***	***SD***	***M***	***SD***	***M***	***SD***
Age (years)	25.9	5.1	22.8	3.4	27.5	5.4	27.6	5.0
Arithmetic ability	14.4	3.6	13.7	3.5	14.8	3.4	14.7	4.0
Sex	38 female; 24 male		14 female; 7 male		12 female; 8 male		12 female; 9 male	

### Arithmetic Tasks

**Figure 1:**
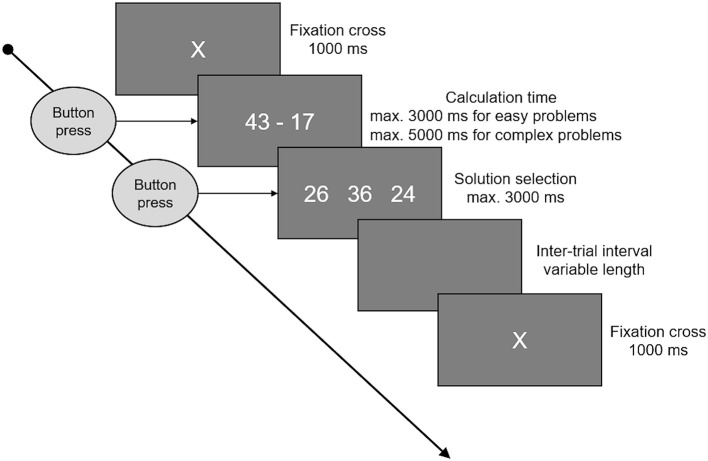
This figure shows the time course of an arithmetic trial. A fixation cross was presented at the beginning, lasting for 1 s. Following the fixation cross, the arithmetic problem was presented on the screen until a button was pressed, indicating that the problem was solved, or time ran out. Maximum time available was 3 s for small and 5 s for large problems. When the button was pressed or time ran out, three options were presented and participants had 3 s to select one of these by pressing the corresponding button. In between the single trials there were inter-trial intervals of variable length, whereby the duration was set to cover the unused time of problem presentation and solution selection, keeping the total trial duration (fixation cross to fixation cross) constant.

Participants were asked to solve three sets of 64 additions and three sets of 64 subtractions. One of each before, another one of each during, and the final sets after stimulation. Each set consisted of 32 small, easy problems, assumed to be solved by fact retrieval, and 32 large problems, assumed to be solved by procedural calculation. The order of the single items was pseudorandomized. Small additions were one-digit/one-digit problems with a maximum sum of 10. The range of possible operands was 2–8. Small subtractions were constructed by mirroring the small additions (3 + 6 = 9 → 9–6 = 3). As these rules result in only 24 possible problems, small problems were the same in all three sets, and in each set eight problems were presented twice. Thereby, the eight repeated problems where different in every set, so that no problem was presented more than four times in total. Large additions were two-digit/two-digit additions with carry, addends between 12 and 59, and sums below 100. Again, the subtractions were constructed by mirroring the additions. Excluding problems with round numbers (e.g., 30) and tie problems, there was a set of 143 large additions/subtractions and 32 of each were randomly selected for each set, without any repetitions. A typical trial is depicted in Figure 1. Every trial started with a fixation cross for 1 s, immediately followed by the arithmetic problem. Problems were presented on screen until the participant pressed a button, indicating she/he had solved the problem. The maximum presentation time (time-out) was three (small problems) or five (large problems) seconds, respectively. After the button was pressed or time ran out, participants had 3 s to choose the correct solution from three options by pressing the corresponding button. To keep trial and set durations constant, a blank screen was presented for the time left from the maximal calculation time and maximal solution selection time before the next trial started. After each set, participants could indicate if they used fact retrieval or procedural strategies more often when processing small, easy problems and separately for large, complex problems. To this end, participants were asked to locate a cursor on a bar ranging from “retrieved” (indicating a 100% retrieval rate) to “calculated” (indicating a 100% procedural calculation rate).

The markers for arithmetic performance were accuracy and calculation times. Accuracy was defined as the percentage of trials solved correctly and in time (before the 3 or 5 s time out) in relation to the total number of trials of this type per set. Calculation time was assessed from start of the problem presentation until the button press, indicating that the participant had solved the problem. Mean calculation times per set (block 1–3 and addition vs. subtraction), and difficulty (small vs. large) were calculated, whereby all incorrect trials or problems not solved in time (i.e., no button press before time out) were excluded.

### Working Memory

**Figure 2:**
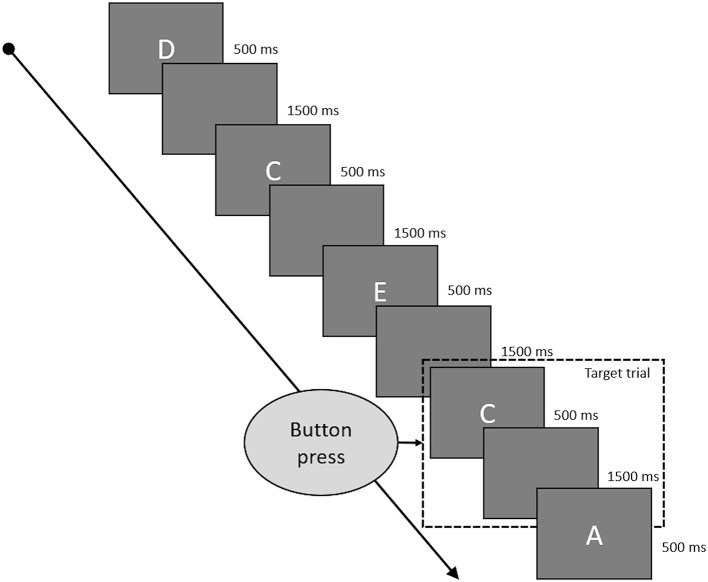
This figure shows some typical trials of the 2-back task. Every letter was presented for 500 ms and followed by a blank screen for 1,500 ms. Target trials were the letters which were identical to the letter presented two trials before. Participants had to press a button if the current trial was a target trial and could do so while the letter was presented to them and during the following blank screen phase.

Working memory was assessed by a letter 2-Back task. Task duration was 180 s with a presentation duration of 500 ms for every letter and 1,500 ms of blank screen between to letters (see Figure 2 for a depiction of the 2-back task). Hence, a single trial was 2,000 ms long, and each run of the 2-back task consisted of 90 trials. The letters used were “A,” “B,” “C,” “D,” and “E,” which appeared in pseudorandomized order to achieve 60 non-target and 30 target trials. Target trials were defined as trials in which the presented letter matched the letter from two trials ago. Participants were instructed to indicate target trials by pressing a button and could do so during the whole trial duration from presentation of one letter to the presentation of the next (500 ms presentation time plus 1,500 ms blank screen). They had to refrain from pressing any button during non-target trials. A correct reaction (CR) consisted of a button press during a target trial, a correct rejection (CRJ) of refraining from a button press during non-target trials. A false alarm (FA) occurred when the button was pressed during a non-target trial and a miss (M) was defined as the absence of a button press during a target trial. Working memory performance was assessed by reaction time (WM-RT) over all correct trials and by accuracy (WM-ACC), calculated by WM-ACC = [1–((FA + M) / 90)) * 100].

### Basic Arithmetic Ability

The subtest “Rechenzeichen” (arithmetic operators) of the IST-2000R (Liepmann et al., 2007) was used as a short assessment of participants' basic arithmetic ability. In this subtest, participants are presented with 20 items consisting of an arithmetic problem and its solution but without the operators (e.g., A ? B ? C = D). They have 10 min to identify the correct operators for the 20 problems. The number of correctly solved problems represents the raw score in this subtest.

### Transcranial Direct Current Stimulation

Stimulation was applied utilizing a NeuroConn DC-Stimulator Plus (NeuroConn, Ilmenau, Germany). Electrodes were rectangular rubber electrodes sized 3 by 3 cm for the anode and 5 by 7 cm for the cathode. The anode was placed over EEG position F3 for frontal stimulation, targeting the left DLPFC, and over P3 for parietal stimulation, targeting the left PPC. The cathode was placed over the contralateral supraorbital site. For half of the participants receiving sham stimulation the electrodes were mounted as for frontal stimulation and for the other half as for parietal stimulation. Electrodes were applied directly to the scalp with an about 1–2 mm thick layer of Ten20 paste (Weaver and Company, Aurora, USA) and held in place by the EEG cap mounted above the stimulation electrodes. In the active stimulation groups, tDCS was applied for 25 min with an intensity of 1 mA and fade in/out phases of 30 s in which the current was slowly ramped up/down. Current density under the electrode was 0.11 mA/cm^2^ for the anode and 0.03 mA/cm^2^ under the cathode. Sham stimulation consisted of a 30 s fade in phase followed by 50 s of applied current (1 mA) and 30 s of fade out, in order to induce the same sensory perception as the active stimulations. Impedances were comparable between the three groups [frontal: *M* = 3.41 kΩ (*SD* = 2.32); parietal: *M* = 3.68 kΩ (*SD* = 2.11); sham *M* = 4.39 kΩ (*SD* = 2.19); *F*_(2, 59)_ = 1.208; *p* = 0.306; $\eta_{\text{p}}^{2}$ = 0.039]. Furthermore, the stimulation was applied in a double-blind way by using the study mode of the DC-Stimulator Plus. Here, a code list was prepared with one code for each subject, and entering the respective code either started active or sham stimulation.

### Electroencephalography

#### Recording

The EEG recording was conducted while the participants processed the arithmetic and working memory tasks in a separate, normally lit and quiet room, using a 64-channel BioSemi ActiveTwo EEG system (BioSemi, Amsterdam, Netherlands). Electrodes were mounted according to the 10:20 system (Jasper, 1958) using BioSemi head caps and Signagel (Parker Laboratories, Fairfield, USA) to ensure appropriate contact. As the tDCS electrodes were mounted at the positions F3, P3, Fp2, and AF8 these EEG electrodes were not mounted.

#### Preprocessing

**Figure 3:**
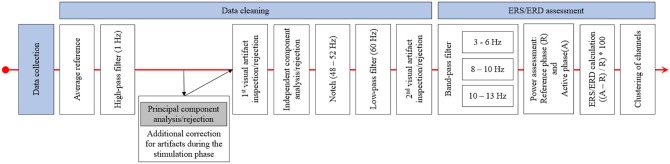
This figure displays the procedure of EEG data pre-processing and ERS/ERD calculation. After data collection, the EEG data was re-referenced and high-pass filtered. This was followed by a principal component analysis for the data recorded during stimulation and by the first visual inspection and rejection of artifacts. The independent component analysis was applied after this first inspection and after bad components were excluded the EEG data was notch and low-pass filtered. The final step was the second visual inspection and artifact rejection. ERS/ERD calculation started with band-pass filtering of the EEG to only include data in the frequency band of interest. Based on this data, the band power in the reference and active intervals was assessed and ERS/ERD values were calculated. The final step was the clustering, by averaging ERS/ERD values over the channels included in the different clusters.

Data was analyzed using MNE (Gramfort et al., 2013, 2014) and additional, custom-built Python code. For the EEG recordings before and after stimulation, pre-processing was done semi-automatically using an average reference, a 1 Hz high-pass filter and visual inspection regarding prominent artifacts and bad channels before applying an independent component analysis (ICA) to remove ocular artifacts. This was followed by applying a notch (48–52 Hz) and a low-pass filter (60 Hz) before a second visual inspection to detect any remaining artifacts. For the EEG data recorded during the stimulation phase, an additional principal component analysis (PCA) step to remove artifacts induced by the stimulator during sham stimulation (repeated impedance checks) was applied before the ICA was performed. Finally, data was prepared separately for each frequency band of interest (theta 3–6 Hz; low alpha 8–10 Hz, and high alpha 10–13 Hz) by applying adequate band-pass filters. The chosen frequency ranges were based on prior studies (Grabner and De Smedt, 2011, 2012). Afterwards, the mean power during the reference interval (R; fixation cross; 1,000 ms) and during activation (A; calculation time from problem onset until button press) in all correct trials consisting of more than 50% artifact-free data was assessed for each frequency band. In each block (before, during, and after stimulation) and arithmetic task (small/large additions/subtractions) on average, *M* = 0.53; *SD* = 1.12 had to be excluded because of artifacts and *M* = 28.56; *SD* = 3.96 trials were used for analysis. The mean power during reference and activation intervals was averaged over all used trials (separately for additions and subtractions as well as for small and large problems), and ERS/ERD values for the four types of arithmetic problems and the three phases were calculated by ERS/ERD = ((A–R) / R) * 100. Hence, positive values indicate ERS (an increase of power from the reference interval to activation) and negative values indicate ERD (a decrease of power from reference interval to activation). Finally, the single channels were grouped into clusters, and single-channel ERS/ERD values were averaged to result in a single ERS/ERD value for each cluster, problem type, and time point (before, during, and after stimulation). The clusters used were left frontal (Fp1, AF3, AF7, F7, FC5, FC3, and FC1), right frontal (AF4, F6, F4, F2, FC6, FC4, FC2), left parietal (CP5, CP3, CP1, P7, PO7), and right parietal (CP2, CP4, CP6, P8, P6, P4, P2, PO8, PO4). In addition to the channels not mounted because of the tDCS electrodes, the channels F1, F5, F8, P1, P5, and PO3 were excluded for all participants, as these channels were closest to the stimulation electrodes and did not yield processable EEG in most of the participants receiving active stimulation. Figure 3 depicts the procedure of EEG data processing.

### Procedure

**Figure 4:**
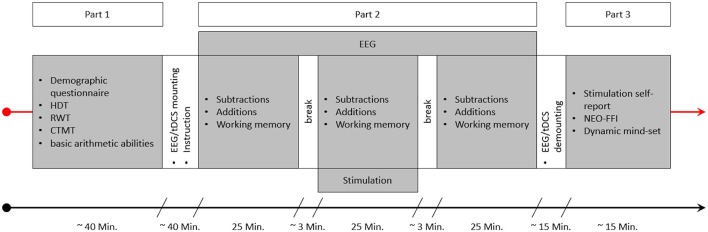
This figure depicts an experimental session. After getting informed about the experiment and signing the consent form the first part of the study started, consisting of a demographic questionnaire, the HDT, RWT, and CTMT, as well as the “Rechenzeichen” (operators) scale of the IST serving as a measure of basic arithmetic ability. This part was followed by the specific instructions for the arithmetic and working memory tasks and the mounting of the EEG and tDCS electrodes. Part 2 consisted of the three blocks of arithmetic and working memory tasks. The order of the three tasks varied between the subjects in a pseudorandomized manner. Stimulation was applied for 25 min during block 2, and EEG was recorded during all three blocks. After part 2 was finished, the EEG and tDCS electrodes were demounted and participants could wash their hair. Finally, in part 3, participants were asked to complete a stimulation self-report, the NEO-FFI, and the dynamic-mindset questionnaire.

The experimental session consisted of three parts (see Figure 4). In the beginning, participants were asked to answer a demographic questionnaire, followed by a short test to ascertain right hand dominance (HDT; Steingrüber and Lienert, 1971), a verbal fluency test (RWT; Aschenbrenner et al., 2000) and the Comprehensive-Trial-Making- Test (CTMT; Reynolds, 2002). The latter two tests were not used in this study, but were part of another project. The last test conducted before the EEG and tES electrodes were mounted was the “*Rechenzeichen” (operators)*-part of the Intelligenz-Struktur-Test 2000R (IST-2000R; Liepmann et al., 2007) as a short assessment of basic arithmetic abilities. Following this, the EEG and tDCS electrodes were mounted and the main test session with EEG recording and stimulation took place. This main test session was the second part of the study and consisted of three blocks, with two sets of arithmetic problems (one containing additions and one subtractions) and one WM task (N-Back task) each. The running order of the arithmetic sets and the WM task was pseudorandomized in order to be balanced over subjects and groups, but was constant for all three blocks (before, during, and after stimulation) within each person (e.g., if for a participant the order was working memory, additions, subtractions in block 1 the tasks would be in the same order in blocks 2 and 3 for this person). The arithmetic sets and the WM task were separated by breaks of 50 s, with longer breaks between the three blocks in order to start and stop stimulation. Before the start of an arithmetic set or the WM task, participants were informed by a sound signal and a message on screen that the tasks will resume, and another message informing them about which task will be next. After the main part was finished, EEG and tDCS electrodes were demounted and participants were asked whether they think they received active or sham stimulation. In the final part, they were asked to answer a stimulation self-report as well as the German version of the NEO-Five-Factor-Inventory (NEO-FFI; Borkenau and Ostendorf, 2008), and a questionnaire regarding their dynamic mind-set, both being part of another project.

### Statistical Analysis

All analyses were conducted using SPSS 25 (IBM, Armonk, USA). Stimulation-induced changes in arithmetic and working memory performance were analyzed using mixed design ANCOVAs with within-subjects factor time (before, during, and after stimulation) and the between-subjects factor treatment (frontal a-tDCS, parietal a-tDCS, and sham stimulation). Analyses were calculated separately for accuracy and calculation time of each arithmetic type (small additions, large additions, small subtractions, large subtractions), and for overall accuracy and reaction time of the WM task. Changes in ERS/ERD values were analyzed by calculating mixed design ANCOVAs with the within-subjects factors time (before, during, and after stimulation), and location (frontal, parietal), and the between-subjects factor treatment (frontal a-tDCS, parietal a-tDCS, and sham stimulation). These analyses were carried out separately for each frequency band and hemisphere, but only for these types of arithmetic problems in which stimulation changed performance, as the main research question regarding the EEG was whether stimulation-induced behavioral changes are reflected in ERS/ERD patterns. Participants' age, sex, and basic arithmetic abilities were used as covariates in all analyses concerning arithmetic performance and related EEG. For analysis of working memory performance, only age and sex were used as covariates. Greenhouse-Geisser correction was used if sphericity could not be assumed as indicated by a significant Mauchly's test of sphericity. Efficacy of blinding was analyzed using a chi-square test on the stimulation self-report data.

## Results

### Behavioral

#### Small Additions

For accuracy in small additions (overall *M* = 98.00%; *SD* = 1.70), the ANCOVA showed no significant main effects of time [*F*_(2, 112)_ = 1.927; *p* = 0.150; $\eta_{\text{p}}^{2}$ = 0.033] or treatment [*F*_(2, 56)_ = 0.157; *p* = 0.855; $\eta_{\text{p}}^{2}$ = 0.006] nor a significant interaction time * treatment [*F*_(4, 112)_ = 0.146; *p* = 0.965; $\eta_{\text{p}}^{2}$ = 0.005].

For calculation times (*M* = 0.75 s; *SD* = 0.13), the ANCOVA showed a significant main effect of time [*F*_(2, 112)_ = 5.792, *p* = 0.004; $\eta_{\text{p}}^{2}$ = 0.094] but no significant effect of treatment [*F*_(2, 56)_ = 2.583; *p* = 0.085; $\eta_{\text{p}}^{2}$ = 0.084] or interaction time * treatment [*F*_(4, 112)_ = 0.471; *p* = 0.757; $\eta_{\text{p}}^{2}$ = 0.017]. Pairwise comparisons showed, that calculation times before treatment (*M* = 0.77 s, *SD* = 0.17) were slower than during treatment (*M* = 0.74; *SD* = 0.14; *p* = 0.009) and after treatment (*M* = 0.73; *SD* = 0.14; *p* = 0.006). Calculation times during and after treatment did not differ (*p* = 0.552).

#### Large Additions

For accuracy in large additions (*M* = 80.63%; *SD* = 8.62), the ANCOVA showed neither significant main effects of time [*F*_(2, 112)_ = 0.215; *p* = 0.807; $\eta_{\text{p}}^{2}$ = 0.004] and treatment [*F*_(2, 56)_ = 0.226; *p* = 0.798; $\eta_{\text{p}}^{2}$ = 0.008] nor a significant interaction time * treatment [*F*_(4, 112)_ = 1.633; *p* = 0.171; $\eta_{\text{p}}^{2}$ = 0.055].

Similarly, for calculation times (*M* = 2.52 s; *SD* = 0.66), the ANCOVA showed neither significant main effects of time [*F*_(1.74, 97.60)_ = 0.419; *p* = 0.631; $\eta_{\text{p}}^{2}$ = 0.007] and treatment [*F*_(2, 56)_ = 0.662; *p* = 0.520; $\eta_{\text{p}}^{2}$ = 0.023] nor a significant interaction time * treatment [*F*_(3.49, 97.60)_ = 1.129; *p* = 0.345; $\eta_{\text{p}}^{2}$ = 0.039].

#### Small Subtractions

For accuracy in small subtractions (*M* = 96.76%; *SD* = 2.89), the ANCOVA showed neither significant main effects of time [*F*_(2, 112)_ = 0.217; *p* = 0.805; $\eta_{\text{p}}^{2}$ = 0.004] and treatment [*F*_(256)_ = 1.089; *p* = 0.344; $\eta_{\text{p}}^{2}$ = 0.037] nor a significant interaction time * treatment [*F*_(4, 112)_ = 0.464; *p* = 0.762; $\eta_{\text{p}}^{2}$ = 0.016].

Similarly as for small additions, the ANCOVA for calculation times in small subtractions (*M* = 0.84 s; *SD* = 0.20) also showed a significant main effect of time [*F*_(2, 112)_ = 5.837, *p* = 0.004; $\eta_{\text{p}}^{2}$ = 0.094] but no significant effect of treatment [*F*_(2, 56)_ = 1.958; *p* = 0.151; $\eta_{\text{p}}^{2}$ = 0.065] or time * treatment interaction [*F*_(4, 112)_ = 0.694; *p* = 0.597; $\eta_{\text{p}}^{2}$ = 0.024]. Pairwise comparisons showed, that calculation times before stimulation (*M* = 0.87 s, *SD* = 0.21) were slower than during stimulation (*M* = 0.84; *SD* = 0.22; *p* = 0.036) and after stimulation (*M* = 0.82; *SD* = 0.22; *p* = 0.003). Calculation times during and after stimulation did not differ (*p* =0.167).

#### Large Subtractions

The ANCOVA showed a significant main effect of time for accuracy (*M* = 74.83%; *SD* = 12.29) in large subtractions [*F*_(2, 112)_ = 12.749; *p* < 0. 001; $\eta_{\text{p}}^{2}$ = 0.185] but no main effect of treatment [*F*_(2, 56)_ = 0.144; *p* = 0.866; $\eta_{\text{p}}^{2}$ = 0.005] or interaction between time and treatment [*F*_(4, 112)_ = 1.406; *p* = 0.237; $\eta_{\text{p}}^{2}$ = 0.048]. Pairwise comparisons showed that accuracy before treatment (*M* = 71.47; *SD* = 15.30) was lower than during treatment (*M* = 76.31; *SD* = 10.77; *p* < 0.001) and after treatment (*M* = 76.71; *SD* = 13.74; *p* < 0.001), while there was no difference between during and after treatment (*p* = 0.708).

**Figure 5:**
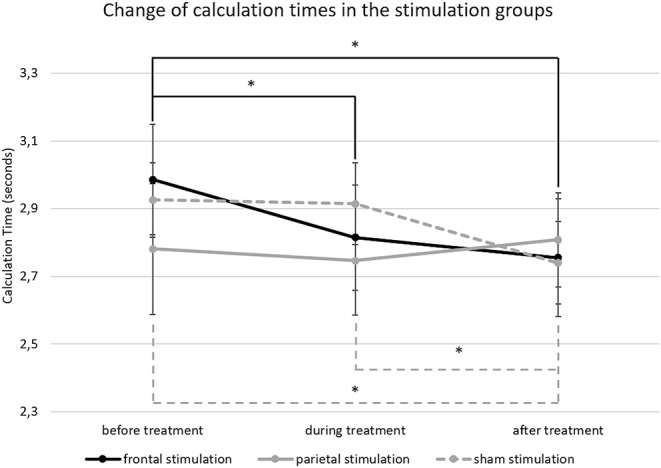
This figure depicts the change of calculation time in large subtractions from before to after treatment. The error bars represent the standard error of the mean and * indicates significant differences (unbroken lines depict differences in the frontal stimulation group and dashed lines in the sham group).

For calculation times in large subtractions (*M* = 2.83 s; *SD* = 0.65), the ANCOVA showed a significant main effect of time [*F*_(2, 112)_ = 5.786; *p* = 0.004; $\eta_{\text{p}}^{2}$ = 0.094) but not for treatment [*F*_(2, 56)_ = 0.096; *p* = 0.909; $\eta_{\text{p}}^{2}$ = 0.003]. However, there was a significant interaction of time * treatment [*F*_(4, 112)_ = 2.787; *p* = 0.030; $\eta_{\text{p}}^{2}$ = 0.091]. Pairwise comparisons showed that the interaction was driven by differences in calculation time reductions over time between the treatment groups. The group receiving frontal a-tDCS showed a significant reduction of calculation times from before treatment (*M* = 2.99; *SD* = 0.75) to during treatment (*M* = 2.81; *SD* = 0.72; *p* = 0.013) and after treatment (*M* = 2.76; *SD* = 0.79; *p* = 0.013) while there was no difference in calculation times between the blocks during and after treatment (*p* = 0.891). The group receiving sham stimulation, on the other hand, showed no reduction of calculation time from before treatment (*M* = 2.93; *SD* = 0.50) to during treatment (*M* = 2.91; *SD* = 0.55; *p* = 0.997), but in the block after treatment (*M* = 2.74; *SD* = 0.56) they were significantly faster than before (*p* = 0.008) and during treatment (*p* = 0.002). Finally, the group receiving parietal a-tDCS showed no differences in calculation times over time (before; *M* = 2.78; *SD* = 0.86; during; *M* = 2.75; *SD* = 0.72; after: *M* = 2.81; *SD* = 0.62; all *p* > 0.05). Results are depicted in Figure 5, and calculation time changes on a single person level are given as additional information in Figure SM1.

#### Working Memory

Regarding WM accuracy, the ANCOVA showed a significant main effect of time [*F*_(2, 114)_ = 5.929; *p* = 0.004; $\eta_{\text{p}}^{2}$ = 0.094] but no effect of treatment (*F*(2,57) = 0.320; *p* = 0.727; $\eta_{\text{p}}^{2}$ = 0.011) or time * treatment interaction [*F*_(4, 114)_ = 1.678; *p* = 0.160; $\eta_{\text{p}}^{2}$ = 0.056]. Pairwise comparisons showed that the accuracy before treatment (*M* = 89.23; *SD* = 5.85) was lower than the accuracy during (*M* = 90.79; *SD* = 6.99; *p* = 0.035) and after treatment (*M* = 91.69; *SD* = 7.02; *p* = 0.004) but did not differ between during and after treatment (*p* = 0.155).

Similarly, for WM reaction times, the ANCOVA showed a significant main effect of time [*F*_(1.767, 100.698)_ = 3.353; *p* = 0.045; $\eta_{\text{p}}^{2}$ = 0.056], but no effect of treatment [*F*_(2, 57)_ = 0.828; *p* = 0.442; $\eta_{\text{p}}^{2}$ = 0.028] or time * treatment interaction [*F*_(3.533;100.698)_ = 0.299; *p* = 0.857; $\eta_{\text{p}}^{2}$ = 0.010]. Pairwise comparisons showed that the reaction times after treatment (*M* = 0.57; *SD* = 0.13) were faster than during treatment (*M* = 0.60; *SD* = 0.13; *p* = 0.002). However, reaction times before treatment (*M* = 0.59; *SD* = 0.13) lay in between the times achieved during and after treatment and did not significantly differ from either (both *p* > 0.05).

### ERS/ERD

#### Theta Band ERS/ERD During Large Subtractions

**Figure 6:**
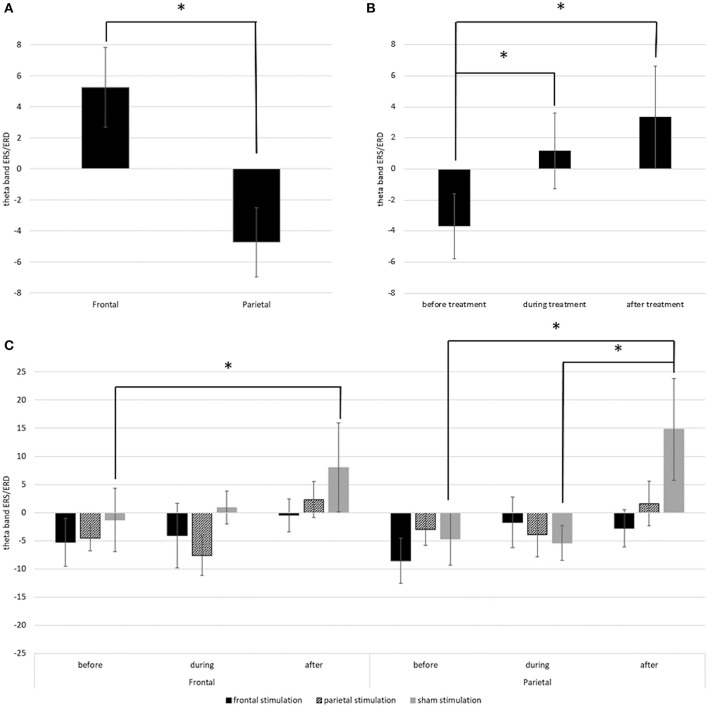
**(A)** Depicts the mean theta band ERS/ERD values in the left frontal and parietal regions over all time points. **(B)** Depicts the overall change of theta band ERS/ERD in the left hemisphere from before to after stimulation. **(C)** Depicts the change of theta band ERS/ERD values in right frontal and right parietal regions in the different treatment groups from before to after stimulation. Error bars represent the standard error of the mean and * indicates significant differences.

**Figure 7:**
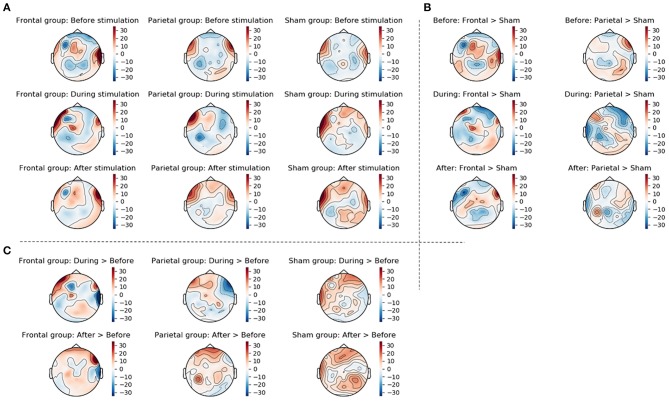
This figure displays the topographic information of average ERS/ERD values in the three groups (frontal stimulation parietal stimulation, sham stimulation) before, during, and after stimulation **(A)**, the difference between frontal and sham groups as well as the difference between parietal and sham groups at the three time points **(B)**, and the change from before to during stimulation and from before to after stimulation in the three groups **(C)**. Changes were calculated by subtracting ERS/ERD values in the sham group from those in the actively stimulated groups for the between group changes displayed in **(B)** and by subtracting the ERS/ERD values before stimulation from those during or after stimulation for the within group changes displayed in **(C)**.

Main results are depicted in Figure 6; additional topographic information containing single channel information is displayed in Figure 7. For the left hemisphere, the ANCOVA showed a significant main effect of time [*F*_(1.437, 80.492)_ = 4.185; *p* = 0.030; $\eta_{\text{p}}^{2}$ = 0.070] and location [*F*_(1, 56)_ = 36.514; *p* < 0.001; $\eta_{\text{p}}^{2}$ = 0.395] but no effect of treatment [*F*_(2, 56)_ = 0.655; *p* = 0.524; $\eta_{\text{p}}^{2}$ = 0.023] or time * location [*F*_(1.603, 89.782)_ = 2.924; *p* = 0.070; $\eta_{\text{p}}^{2}$ = 0.050], time * treatment [*F*_(2.875, 80.492)_ = 0.506; *p* = 0.671; $\eta_{\text{p}}^{2}$ = 0.018], location * treatment [*F*_(2, 56)_ = 1.127; *p* = 0.331; $\eta_{\text{p}}^{2}$ = 0.039], and time * location * treatment interactions [*F*_(1.603, 89.782)_ = 1.771; *p* = 0.140; $\eta_{\text{p}}^{2}$ = 0.059]. Overall, participants showed a theta band ERS over frontal regions (*M* = 5.26; *SD* = 20.26), but an ERD over parietal regions (*M* = −4.74; *SD* = 17.50) with a decrease of ERD / increase of ERS over time (Figures 5A,B). Thereby, before treatment, participants showed a theta band ERD (*M* = −3.69; *SD* = 16.56) which was significantly different from the values during (*M* = 1.16; *SD* = 19.23; *p* = 0.042) and after treatment (*M* = 3.31; *SD* = 26.14; *p* < 0.001) where participants showed ERS patterns. ERS values during and after treatment did not differ (*p* = 0.494).

For the right hemisphere, the ANCOVA showed significant main effect of time [*F*_(1.574, 88.162)_ = 7.070; *p* = 0.003; $\eta_{\text{p}}^{2}$ = 0.112] and a significant time * location * treatment interaction [*F*_(3.918, 109.692)_ = 3.293; *p* = 0.014; $\eta_{\text{p}}^{2}$ = 0.105]. Effects of location [*F*_(1, 56)_ = 0.017; *p* = 0.898; $\eta_{\text{p}}^{2}$ = 0.000] and treatment [*F*_(2, 56)_ = 0.752; *p* = 0.476; $\eta_{\text{p}}^{2}$ = 0.026] and the interaction time * location [*F*_(1.959, 109.692)_ = 0.668; *p* = 0.515; $\eta_{\text{p}}^{2}$ = 0.012], time * treatment [*F*_(3.148, 88.162) =_ 0.901; *p* = 0.448; $\eta_{\text{p}}^{2}$ = 0.031], and location * treatment [*F*_(2, 56)_ = 0.272; *p* = 0.763; $\eta_{\text{p}}^{2}$ = 0.010] were not significant. Pairwise comparisons showed, that the interaction time * location * treatment was driven by the sham stimulated group. In this group, there was a significant difference in ERS/ERD values over frontal regions between before (*M* = −0.84; *SD* = 36.54), and after treatment (*M* = 7.53; *SD* = 42.68; *p* = 0.033) while the ERS/ERD values in the active stimulation conditions did not differ between the timepoints (both *p* > 0.05). Over parietal regions, the sham group also showed a significant change in ERS/ERD values from before (*M* = −4.46; *SD* = 31.59) to after treatment (*M* = 14.80; *SD* = 49.39; *p* < 0.001), and additional differences between the ERS/ERD values during treatment (*M* = −5.35; *SD* = 31.79) and after treatment (*p* = 0.001) but no difference between ERS/ERD values before and during treatment (*p* = 0.829). All other pairwise comparisons were non-significant (*p* > 0.05).

#### Low Alpha Band ERS/ERD During Large Subtractions

For the left hemisphere, the ANCOVA showed a significant effect of locations [*F*_(1, 56)_ = 36.806; *p* < 0.001; $\eta_{\text{p}}^{2}$ = 0.397] with a stronger ERD in parietal regions (*M* = −31.63; *SD* = 21.26) than in frontal regions (*M* = −13.68; *SD* = 35.23). No other main effects or interactions were significant (all *p* > 0.05).

Similarly, for the right hemisphere, the ANCOVA also only showed a significant effect of location [*F*_(1, 56)_ = 10.729; *p* = 0.002; $\eta_{\text{p}}^{2}$ = 0.161], with stronger ERD in parietal regions (*M*= −26.49; *SD* = 29.27) than in frontal regions (*M* = −21.26; *SD* = 27.66). All other main effects and interactions were non-significant (all *p* > 0.05).

#### High Alpha Band ERS/ERD During Large Subtractions

For high alpha, the ANCOVA also showed a significant main effect of location in the left hemisphere [*F*_(1, 56)_ = 16.414; *p* < 0.001; $\eta_{\text{p}}^{2}$ = 0.227], with a stronger ERD in parietal regions (*M* = −26.16; *SD* = 17.77) as compared to frontal regions (*M* = −17.89; *SD* = 22.28). Again, all other main effects and interactions were non-significant (all *p* > 0.05).

This also holds true for the right hemisphere, were, again, only the main effect of location proved significant [*F*_(1, 56)_ = 18.199; *p* < 0.001; $\eta_{\text{p}}^{2}$ = 0.245], with a stronger ERD in parietal regions (*M* = −24.48; *SD* = 19.71) than in frontal regions (*M* = −18.37; *SD* = 21.13). All other main effects and interactions were non-significant (all *p* > 0.05).

### Blinding

A chi-square test showed no relation between the subjective perception and actually applied stimulation [active vs. sham; χ^2^(2, *N* = 62) = 2.357; *p* = 0.308]. In the frontally stimulated group, there were 13 participants assuming to have received active stimulation and 8 assuming to have received sham stimulation. In the parietally stimulated group this ratio was 8–12 and in the sham group 9–12.

## Discussion

The aim of the present study was to extend prior research by directly comparing the effects of a-tDCS over left frontal (targeting the DLPFC) and left parietal (targeting the PPC) regions on arithmetic performance in small and large problems of different operations and on EEG activity. In order to be able to conduct a more fine-grained analysis, performance and EEG were assessed before, during, and after stimulation, allowing for a separation of online and after-effects of stimulation on both, the behavioral and the neurophysiological level. Additionally, WM performance was assessed to investigate whether the effects of a-tDCS on arithmetic performance are task specific. Overall, we found no general tDCS related improvements in arithmetic or working memory performance, but there is some evidence for an acceleration of training gains in participants receiving frontal stimulation. These participants showed a significant improvement in calculation times in large subtractions from before to during and after stimulation, while participants receiving sham stimulation showed a similar change only in the last block. However, this admittedly small effect was not reflected in ERS/ERD patterns.

### Behavioral Effects

Contrary to the expectations and results in prior literature (Hauser et al., 2013; Pope et al., 2015; Rütsche et al., 2015), neither left frontal, nor left parietal a-tDCS induced a general improvement in arithmetic performance. While participants did show some performance improvements over time in all but one type of arithmetic problems (in large additions no improvements emerged), these were mostly general improvements in accuracy (large subtractions) or calculation times (small additions) that can be attributed to practice effects. However, in large subtractions, left frontal a-tDCS led to an accelerated improvement in calculation times from before to during stimulation, as compared to the group receiving sham stimulation. The group receiving sham stimulation also improved, but later on, and the group receiving a-tDCS over parietal regions showed no improvement in calculation times over time. The earlier reduction of calculation times in the frontally stimulated group might indicate accelerated training gains. Although large problems were not repeated, the procedural strategies used to solve them have been trained over the course of the three arithmetic blocks, as can be seen from the general improvement in accuracy and the gains in calculation times in the frontally and sham stimulated groups. Stimulation effects might have not been strong enough for an overall performance improvement, but sufficient to support training gains. This is in line with a recent meta-analysis showing that tDCS effects on training or learning gains are generally larger than those on performance (Simonsmeier et al., 2018).

The absence of any improvement in the group receiving left parietal a-tDCS is startling, as it was parietal a-tDCS that showed promising effects on calculation times in large arithmetic problems (Rütsche et al., 2015) and specifically large subtractions (Hauser et al., 2013) in earlier studies. On the other hand, the detrimental effects of parietal a-tDCS on the accuracy in small arithmetic problems reported by Rütsche et al. (2015) also could not be replicated. All groups showed very fast calculation times and high accuracies in small additions as well as small subtractions and performance improved over time without any stimulation induced differences. One possible explanation for these diverging results could be found in the difference of the timing of tDCS between this study and prior studies finding positive effects (Hauser et al., 2013; Pope et al., 2015; Rütsche et al., 2015). In these prior studies, stimulation was applied before or in between two sets of the arithmetic tasks, while in the present study stimulation was applied concomitant to the task. There is some evidence for differences in stimulation effects depending on preexisting activity, indicating a neuronal state dependency of non-invasive brain stimulation effects in general (Silvanto et al., 2008; Romei et al., 2016). Additionally, there are other slight differences to prior studies like electrode size (smaller in the present study), positioning of the anode (P3 in the present study but P5/CP5 in Rütsche et al., 2015), or stimulation intensity (1 mA in the present study but 2 mA in Pope et al., 2015), which might have contributed to the differences in results. Furthermore, calculation times were faster in the present study as compared to prior ones (Hauser et al., 2013; Rütsche et al., 2015). This might have been brought about by the time limits for calculations (3 sec. for small; 5 sec. for large problems) which were, together with the inter-trial intervals, implemented to keep the set durations constant. These limits could have induced some time pressure leading to a faster processing of the tasks and hence, less scope for further improvements by stimulation, especially in large problems. Finally, the question regarding the task specificity of tDCS, whether arithmetic processes are improved directly or indirectly by beneficial effects of tDCS on working memory, remains ambiguous. Not only were tDCS effects on arithmetic performance limited to one task and rather small, but, contrary to prior results (Brunoni and Vanderhasselt, 2014), results show no tDCS related effects on working memory.

### Electrophysiological Effects

Since on a behavioral level there only was a small stimulation effect on training gains in large subtractions, the focus regarding neurophysiological changes was set to ERS/ERD patterns emerging during this type of problem. However, results showed no clear stimulation related effects. Interestingly, there was no change in low and high alpha ERD patterns in general, although there was a general improvement in performance in large subtractions.

There were, however, some interesting changes in theta band ERS/ERD patterns accompanying the processing of large subtractions over time. In the left hemisphere, there was a general change from an ERD pattern during block 1 to an ERS pattern during block 3. As in the area of mental arithmetic theta band ERS has been associated with the cognitive less demanding fact retrieval process (De Smedt et al., 2009; Grabner and De Smedt, 2011) and fact training led to an increase in theta band ERS (Soltanlou et al., 2019), this general increase in theta band ERS could reflect the training effect. However, as in this study, the procedural problems were never repeated and hence, no fact training existed, it could only reflect a decrease in cognitive demand of the increasingly trained procedural calculation process. Another probably more plausible explanation could be that this change reflects an increasing demand on attentional processes and cognitive control. Several studies found that frontal theta ERS is also associated with these processes (Missonnier et al., 2006; Cavanagh and Frank, 2014; Ishii et al., 2014). All three blocks consisted of a WM part and two sets of arithmetic problems, which were only separated by short breaks. As the duration increases, the demand on attentional and control processes to carry out the tasks could have increased and hence, led to an increase in associated theta band ERS. The stronger ERS in frontal regions as compared to parietal regions supports this notion.

A similar pattern was also found in the right hemisphere, but only in the group receiving sham stimulation. While this could be a fortuitous effect, it also could indicate an effect of stimulation. Anodal tDCS has been shown to induce wide spread effects and modulate activity in broad networks and different sites of the brain (Polanía et al., 2011; Pena-Gomez et al., 2012). In this case, anodal stimulation of left hemispheric sites of the brain could have modulated activity in right hemispheric regions via mechanisms of interhemispheric inhibition and hence, might have hindered a similar theta band ERS increase as seen in the sham group. However, this explanation can so far only be speculative, and further research is needed to investigate such effects, especially as there were no effects in the stimulated sites themselves.

Another possible explanation could be that the absence of theta band ERS changes in the right hemisphere of the stimulated groups is caused by the cathodal return electrode. This is also one of the limitations of this study. The cathode was mounted at the contralateral supraorbital site. Although a larger electrode (5 × 7 cm) was used, rendering the applied current density beneath it rather low (0.03 mA/cm^2^), an inhibitory effect of this electrode on right frontal areas, especially the frontopolar area, cannot be ruled out completely. This could have disturbed the theta band ERS change in the right hemisphere, at least in frontal regions. However, both explanations come short in explaining why both stimulated groups (left frontally and left parietally) show an absence of theta band ERS increase in right frontal and right parietal sites as compared to the sham group. Another possible issue brought about by the cathode is its potential impact on behavioral effects. As frontopolar regions have been thought of as a metacognitive hub-region (Burgess and Wu, 2013), important for cognitive processes in general, inhibitory effects induced by the cathode might have prevented stronger effects of the anodal tDCS over left frontal and parietal sites. Other studies used a larger cathodal electrode (Rütsche et al., 2015; e.g., 10 × 10 cm in Hauser et al., 2013), or used an extracephalic return electrode (Pope et al., 2015), which might have mitigated or prevented similar disadvantageous effects.

A second limitation is that in this study a forced choice format (participants chose their answer from three options) was used. While this was similar to the work of Hauser et al. (2013) other groups like Rütsche et al. (2015) required the production of answers. This might be a reason why Rütsche and colleagues found detrimental effects of stimulation on the accuracy in easy problems, while this study did not. The task format used in the current study might have allowed the participants to reconsider their answer in light of the displayed options. However, the comparably high accuracy in small problems in this study (additions *M* = 98.00%; subtractions *M* = 96.76%) and in the study of Rütsche et al. (2015); stimulated *M* = 97.82%; sham *M* = 98.78%) speaks against this notion.

In conclusion, neither left frontal, nor left parietal stimulation led to a general improvement of arithmetic or working memory performance. However, there was a significant stimulation effect indicating an acceleration of training gains in large subtractions by left frontal stimulation. As stimulation effects on training and learning seem to be stronger than on performance *per se* (Simonsmeier et al., 2018), the effects might have been too small to enhance performance but still strong enough to improve procedural training. Hence, tDCS might be suited best to improve later performance when applied during learning or training while its potential to improve skills or their application in the sense of a sole performance enhancer remains ambiguous.

## Data Availability Statement

The datasets generated for this study are available on request to the corresponding author.

## Ethics Statement

The studies involving human participants were reviewed and approved by Ethics Committee at the University of Graz, University of Graz, Graz, Austria. The patients/participants provided their written informed consent to participate in this study.

## Author Contributions

JM, CB, MN, and RG: study conception and design. JM and CB: data collection and analysis. JM, CB, MN, and RG: writing and/or critically revising the manuscript.

### Conflict of Interest

The authors declare that the research was conducted in the absence of any commercial or financial relationships that could be construed as a potential conflict of interest.
