# Supplementary material for: Effects of Anodal tDCS on Arithmetic Performance and Electrophysiological Activity
Source: Front Hum Neurosci. 2020 Feb 11;14:17. doi: 10.3389/fnhum.2020.00017 (PMC7026470; doi:10.3389/fnhum.2020.00017)
Supplement: Supplementary file 1 [file Table_1.DOCX]

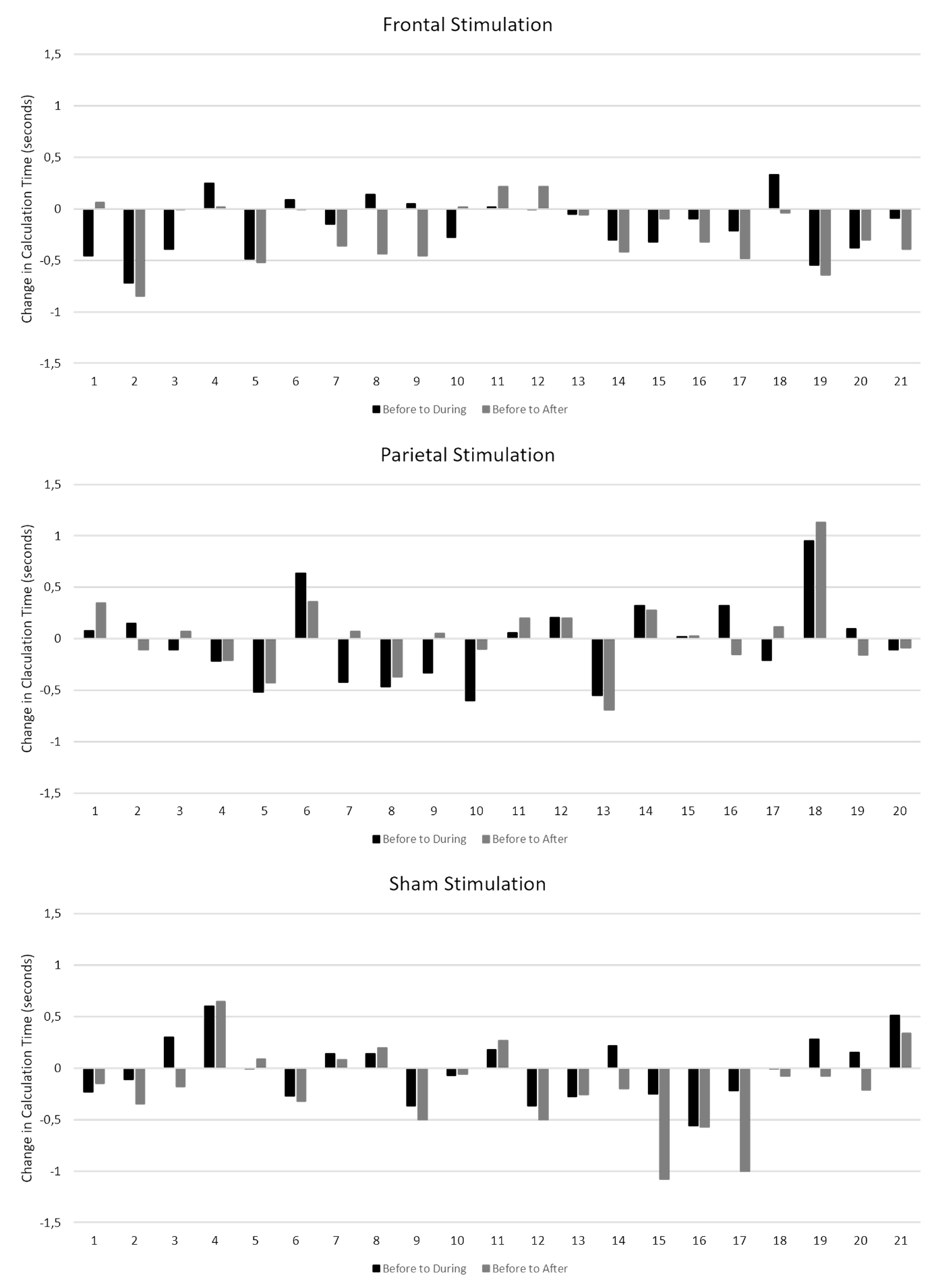
Figure SM1:

*Figure SM1 displays the change in calculation time in large subtractions from before to during stimulation (black bars) and from before to after stimulation (grey bars) for each subject in each group. Values are changes in seconds and were calculated by subtracting the calculation times before stimulation from those during and after stimulation. Hence, negative values indicate a decrease in calculation times over time and positive values an increase.*
